# Feasibility and acceptability of a novel biomedical device to prevent neonatal hypothermia and augment Kangaroo Mother Care in Kenya: Qualitative analysis of focus group discussions and key Informant Interviews

**DOI:** 10.1371/journal.pgph.0001708

**Published:** 2024-04-16

**Authors:** Nudar A. Bhuiya, Scott Liu, David Muyodi, Sherri L. Bucher

**Affiliations:** 1 Scholarly Concentration in Public Health Certificate Program, Indiana University School of Medicine and Richard M. Fairbanks School of Public Health, Indiana University–Indianapolis, Indianapolis, Indiana, United States of America; 2 Moi Teaching and Referral Hospital, Eldoret, Kenya; 3 Department of Community and Global Health, Richard M. Fairbanks School of Public Health, Indiana University—Indianapolis and Department of Pediatrics, Division of Neonatal-Perinatal Medicine, Indiana University School of Medicine, Indianapolis, Indiana, United States of America; Jhpiego, UNITED STATES

## Abstract

Hypothermia is a leading newborn complication, especially among premature and/or low birth weight infants. Within low/middle-income countries where incubators and radiant warmers are often in short supply, leading to gaps in the thermal care chain, neonatal hypothermia underlies high rates of newborn morbidity and mortality. Kangaroo Mother Care/Skin-to-skin care is an effective method for prevention of hypothermia in premature and low birthweight babies but can be very burdensome for families and healthcare providers. Our international multidisciplinary team has developed a prototype for a wearable biomedical device (“NeoWarm”) to provide continuous thermal care and augment kangaroo mother care practices in low-resource settings. The objective of this study was to assess the feasibility and acceptability of NeoWarm and to obtain user design feedback for an early prototype from among adult end-users in Western Kenya. We performed key informant interviews (n = 17) among healthcare providers and 5 focus group discussions (FGDs) among 3 groups of adult stakeholders of premature babies, including: (1) parents/family members of premature babies aged 6 weeks or less (3 FGDs); (2) healthcare providers of newborns (e.g., nurses; physicians; 1 FGD); (3) community opinion leaders and stakeholders (e.g., traditional birth attendants; pastors; village elders; 1 FGD). Content and thematic analyses of transcripts indicate that NeoWarm is acceptable and feasible in promoting facility-based kangaroo mother care in the Kenyan setting. Novel findings derived from respondents include (1) the ability of the device to potentially overcome several barriers to traditional kangaroo mother care methods and (2) user-driven encouragement to expand the use case of the device to potentially include community-based kangaroo mother care and neonatal transport. User design feedback obtained during the interviews informed several key design iterations for subsequent prototypes of the device.

## Introduction

There are an estimated 15 million premature births every year. Globally, complications from prematurity are one of the three leading causes of newborn and under-5 years mortality.[1, 2] Neonatal hypothermia, the inability of a newborn to regulate their body temperature at or above 36.5°C, is an extremely common complication among prematurely born (prior to 37 weeks gestation) and low birthweight (LBW; less than 2500 grams) infants,[3, 4] and plays a significant role in neonatal deaths.[5] Premature and LBW babies are particularly prone to hypothermia due to various factors such as decreased subcutaneous fat, high body water content, immature skin, and large surface area-to-body ratio.[6] Thermal care is therefore an essential part of newborn management and can avert up to 40% of neonatal deaths.[7]

Kangaroo Mother Care/Skin-to-Skin care (KMC/STS) is an evidence-based newborn care intervention[8] that has been demonstrated to ameliorate neonatal hypothermia.[9] Other benefits of KMC include a decrease in infant mortality,[10] reduced risk of sepsis,[9] better weight gain in the newborn,[11] maternal bonding and attachment,[12] early initiation of breastfeeding,[13] and reduced length of hospital stay.[14] As compared to conventional thermal care via incubator or radiant warmer and followed by KMC/STS, immediate KMC/STS, implemented prior to medical stabilization, has been shown to significantly reduce the risk of death in neonates < 1.8 kg in the first 28 days (RR = 0.75, (95% CI 0.64 to 0.89, P = 0.001).[15]

Despite the many demonstrated benefits of KMC/STS, uptake, adoption, scale-up, and compliance remain low in some low/middle-income countries (LMICs) where rates of neonatal mortality due to complications from prematurity are the highest. This is due to numerous barriers, bottlenecks, and gaps across domains (e.g., sociocultural; health service; behavioral) within the implementation context.[16–20] Among some of the key challenges that have been identified to the adoption and scale-up of KMC/STS in LMICs include lack of sufficient hospital staff [21] and low maternal compliance [22]. Within the Kenyan setting, severe shortages of health workers, particularly those with specialized newborn care training, is a chronic problem which often leads to missed nursing care,[23–26] including gaps in provision of thermal care [27].

Our multidisciplinary international team has developed a biomedical device that is designed to prevent neonatal hypothermia among premature/low birthweight babies, while simultaneously addressing some of the known challenges and barriers to the consistent implementation [28] of KMC/STS in LMICs. We hypothesize that incorporating this biomedical device into existing newborn care efforts will improve the adoption of, and compliance to, facility-based KMC/STS. We have developed a prototype of the biomedical device, called “NeoWarm,” and performed engineering bench verification for features such as automated detection of body temperature, self-warming, and vital signs monitoring [29, 30]. The purpose of the current study is to report findings regarding feasibility and acceptability of an early prototype of the device from among adult stakeholders of prematurely born and small babies in Kenya. In addition, we elicited user-design feedback from Kenyan healthcare providers, parents and family members, and community stakeholders.

## Methods

### Description of the NeoWarm biomedical device

**Fig 1:**
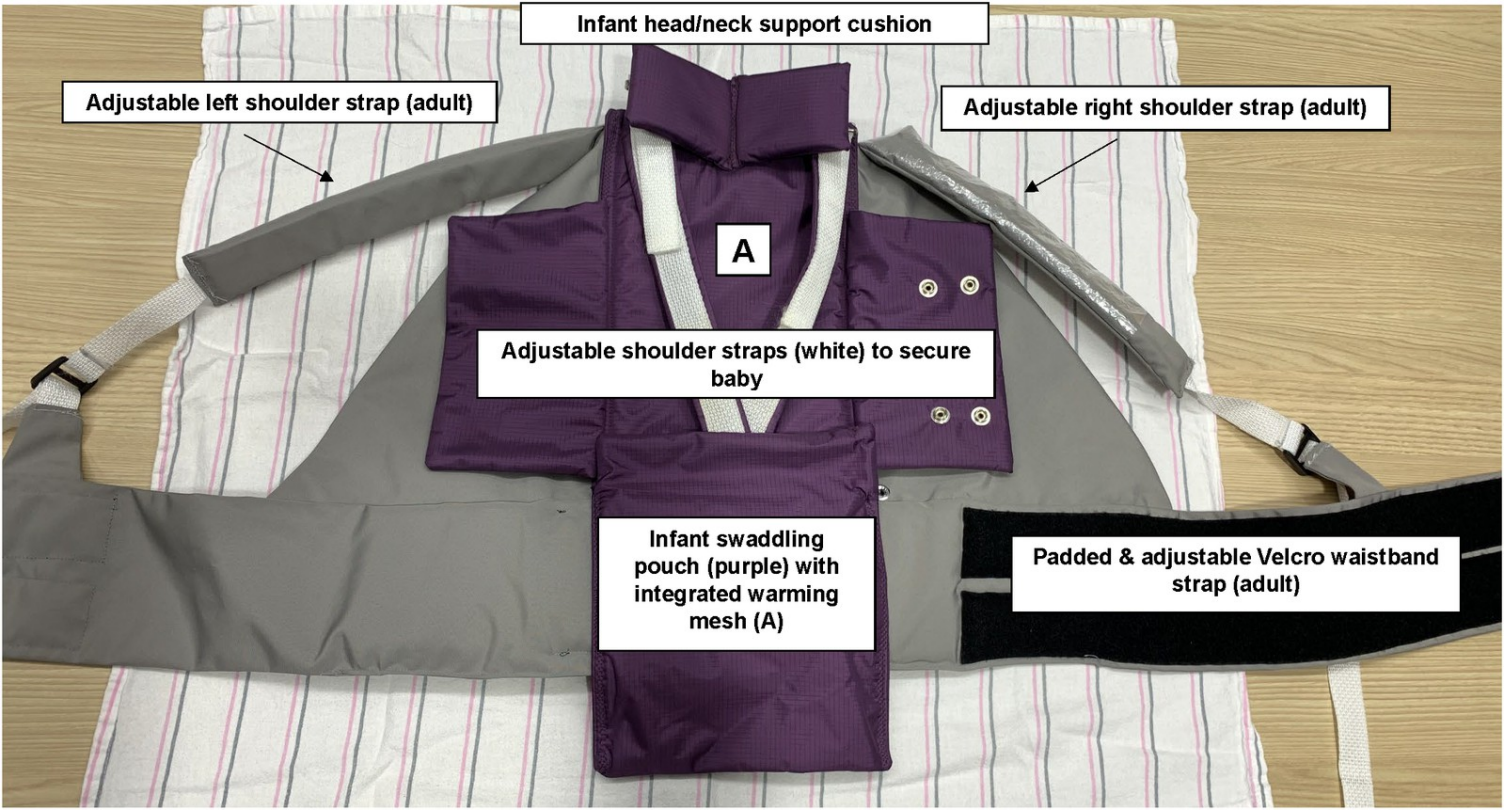
Biomedical device (“NeoWarm”) Built prototype (v1.01) of a biomedical device composed of a purple self-warming swaddling pouch with flaps that open for KMC/STS (worn by baby) + Attached gray carrier (worn by adult during KMC/STS). Self-warming (A) and sensor technology is built into the device to automatically detect & continuously monitor neonatal body temperature. Patents include: [31–33].

**Fig 2:**
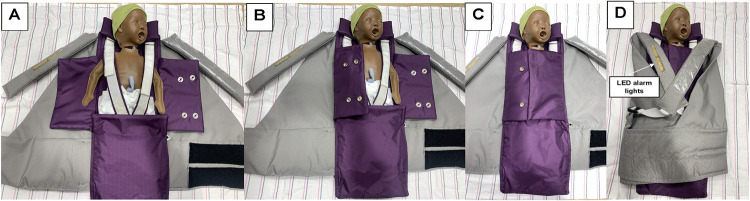
NeoWarm stand-alone mode Panel 2A shows a neonatal simulator “PreemieNatalie,”[38] positioned in the interior of the NeoWarm device with the swaddling pouch flaps open; Panel 2B depicts the first flap being closed; Panel 2C demonstrates the second flap closed, and snapped into position; Panel 2D depicts the gray carrier (worn by an adult caregiver during KMC/STS mode) being wrapped in such a way as to support an on-the-back safe sleep position, and allow adult caregivers to monitor the strip of LED lights (gray shoulder strap) that indicate whether or not an infant is within the normal body temperature range.

NeoWarm is a patented biomedical device (“NeoWarm”, Fig 1 [29, 31–33]) which is a carrier and integrated self-warming swaddling pouch, designed to prevent neonatal hypothermia and facilitate the adoption of, and compliance to, KMC/STS. This study describes feasibility, acceptability, and user-design feedback that was elicited for an initial built-prototype (v1.01) in 2016. As shown in Fig 2, the integrated prototype was composed of a swaddling “pouch” with 2 flaps.

These flaps snapped shut to envelope the baby and prevent heat loss when the device was in “stand-alone” mode (i.e., when the adult-baby dyad was not engaged in KMC/STS; Fig 2). While “continuous” KMC/STS (i.e., 24 hours a day of uninterrupted skin-to-skin contact) is often the recommended best practice for small babies, pragmatically, this standard is often very difficult to achieve–in some cases, due to pain and fatigue among maternal caregivers, lack of availability of health workers and/or family members to provide on-going support, and various health system factors.[34–36] More commonly, “intermittent” KMC/STS is practiced [37]. Thus, the stand-alone mode was developed to ensure that, even when an adult caregiver had to take a break from KMC/STS, for example, to attend to personal needs (e.g., bathe) or rest, that the infant was still receiving seamless thermal support, even in environments with a paucity of incubators.

**Fig 3:**
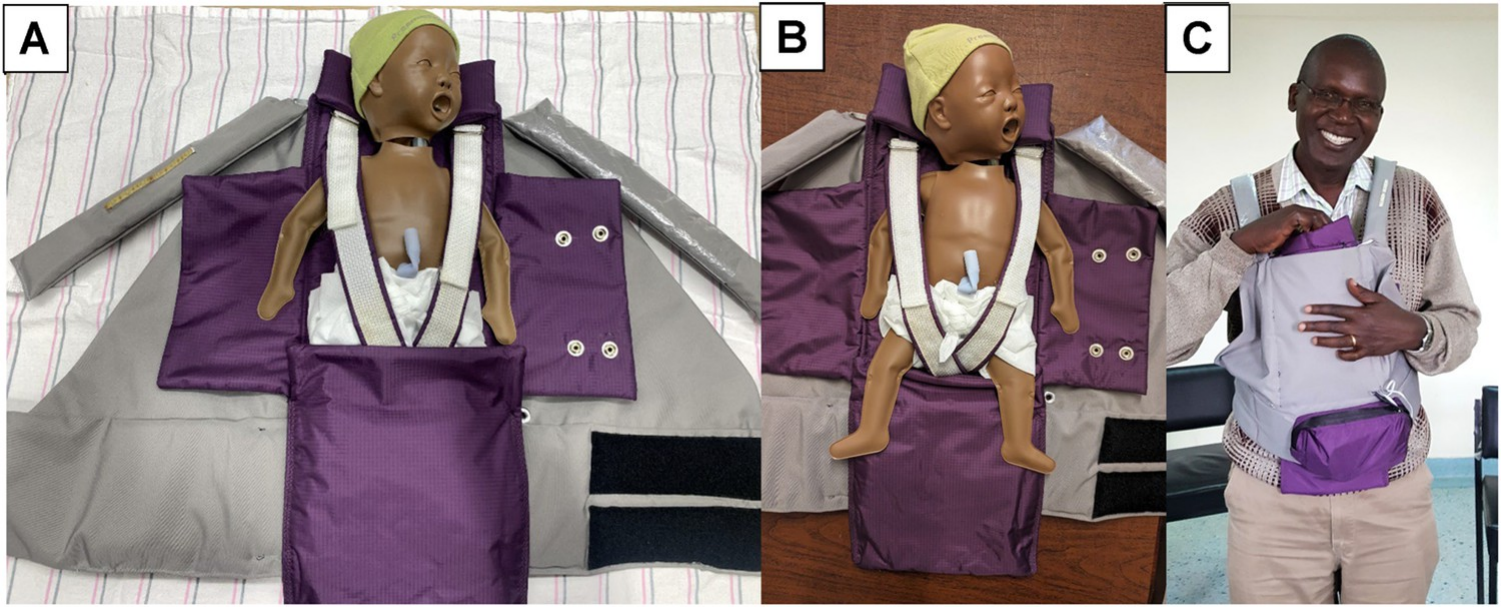
NeoWarm KMC/STS mode Panel 3A depicts the PreemieNatalie simulator in the NeoWarm device with the swaddling pouch flaps open, and the baby’s legs tucked into the bottom of the pouch; Panel 3B: prior to donning the device for KMC/STS, the infant’s legs are removed from the bottom section of the swaddling pouch, so that the “flexed frog” position can be achieved; Panel 3C: One of the co-authors, DM, demonstrates the NeoWarm prototype, with the PreemieNatalie simulator, when being worn in KMC/STS mode. Please note that for demonstration purposes, DM did not remove his sweater or open his shirt. However, in actual use during KMC/STS mode, the adult caregiver will be bare-chested.

When the adult caregiver and premature baby are engaged in KMC/STS, the flaps of the purple swaddling pouch open, allowing for skin-to-skin (chest-to-chest) contact between the infant and the adult, the latter of whom secures the baby in the KMC/STS position by donning the gray carrier (Fig 3). The version 1.01 NeoWarm prototype integrated sensor, circuit board, and microcontroller technologies to automatically detect and continuously monitor neonatal body temperature during 3 potential modes of operation. These modes included: (a) stand-alone (Fig 2); (b) KMS/STS (both described previously and depicted in Fig 3); (c) breastfeeding (not depicted). During breastfeeding mode, the maternal caregiver simply slips one strap off her shoulder and positions the baby to feed. During this mode of operation, the mother can choose whether to have the flaps of the purple swaddling pouch opened, to maintain STS contact while breastfeeding, or closed, to prevent potential heat loss.

The 1.01 prototype was configured with both visual and audio alarms. There was a strip of 3 LED diode lights on the front shoulder strap of the carrier (see Fig 2D and Fig 3C). The device was programmed so that the acceptable body temperature range was configured to be 36.5°C– 37.5°C. If the device detected that the neonate’s body temperature was within this range, then the self-warming mechanism (a flexible heating mesh integrated into the purple swaddling pouch; Fig 1) was “off,” and a green LED diode on the strap of the carrier illuminated. It the device detected a neonatal body temperature of 37.6°C or above, the self-warming mechanism remained “off;” a red LED light illuminated, and an auditory alarm sounded. If the device detected a newborn body temperature of 36.4°C or below, the warming element would engage, and a blue LED light illuminated. If, after 10 minutes of the warming element being engaged, the baby’s body temperature had not risen to 36.5°C or above, then an audio alarm, with tone and cadence distinct from that of the “too hot” alert, sounded.

### Theoretical framework and conceptual approach

Our approach to the design and development of innovative tools and solutions to reduce neonatal mortality is rooted within the theoretical approach of implementation science [39, 40], and utilizes a health services strengthening conceptual framework and community-based participatory research methodology [41] for the targeted integration and scale-up of high-impact, evidence-based health interventions into health systems [42–44]. Our technological innovations are firmly nested within a comprehensive, multi-faceted, integrated approach to the problem of newborn hypothermia and care for premature/small babies. Our bioengineering development processes utilize a Human/User-centered approach and incorporate principles of Design Thinking. Participatory community engagement is a key aspect of all of our global health research and implementation initiatives [45–47].

### Ethical approval

Ethical approval was provided by the Indiana University Institutional Review Board (Exempt approval #1602698245) and Moi University Institutional Review Ethics Committee (IREC/2016/19 - #0001660) All participants provided written informed consent to participate in the study, including to be audio and/or videotaped. They agreed that de-identified photos and video and audio clips could potentially be utilized for scientific or educational purposes.

### Study setting, design, and participants

This qualitative study was conducted in Kenya, in collaboration with the Moi University College of Health Sciences and Moi Teaching and Referral Hospital in Eldoret (Uasin Gishu County), and within selected rural communities and health facilities in western Kenya (Alupe; Busia County and Bokoli; Bungoma County) which are part of the IU-Kenya’s *Global Network for Women’s and Children’s Health Research* Maternal Newborn Health Registry [48–50].We sought feasibility, acceptability, and user design feedback from 3 key stakeholder groups within a newborn’s ecosystem. These include: (1) parents (18 years or older) of premature infants less than 6 weeks of age and their family stakeholders (e.g., grandmothers; grandfathers; adult siblings); (2) maternal and newborn care health workers at facilities with and without formal KMC/STS wards; (3) community stakeholders and opinion leaders (e.g., traditional birth attendants; community health workers; pastors; women’s group representatives; village elders).

Purposive convenience sampling was utilized. Potential subjects who fit the eligibility criteria were identified from within Moi Teaching and Referral Hospital (Eldoret), or from health facilities and communities in western Kenya within the Alupe and Bokoli Maternal Newborn Health Registry clusters. Most of the key informant interviews with healthcare providers were conducted February 11–17, 2016 (N = 11), with additional KIIs (n = 6) and all 5 FGDs conducted November 17 –December 2, 2016. The data collection cycle was influenced, in part, by the US-based Principal Investigator’s (SLB) scheduled research travel to and from Kenya.

### Data collection

Detailed information regarding the composition of the research team and reflexivity is found in S1 Appendix Research Team and Reflexivity. Upon recruitment to participate in the study, each participant was privately consented. Only persons who provided written consent to participate in the study were then scheduled to attend either an interview (healthcare providers only) or focus group discussion. All participants received a copy of their signed study information sheet. None of the persons deemed eligible to participate in the study refused; all provided both written consent at recruitment, and verbal re-consent immediately prior to the KII or FGD (S1 Appendix Research Team and Reflexivity).

Each of the KIIs and FGDs began with a review of the Study Information Sheet and an opportunity for participants to ask questions or seek additional clarification. Semi-structured KIIs and FGDs were co-facilitated by SLB and DM, utilizing a Facilitator Guide that was approved by Indiana University IRB and Moi University IREC (S2 Appendix Interview Guide). Each session was audio and videotaped. These audio and video recordings were immediately uploaded to a password protected laptop of SLB, then erased from the recording devices.

Key Informant Interviews typically lasted 30–60 minutes, and FGDs ranged from 1.5–2 hours. For demonstration of the NeoWarm biomedical device and user-design feedback sessions, a water-filled newborn simulator, from Laerdal Global Health, was utilized. The “PreemieNatalie” simulator is a realistically sized newborn care training mannikin which approximates the weight (1.6 kilograms) and size of a 32 week gestational newborn [38]. Participants in KIIs and FGDs had ample opportunity to try on the NeoWarm prototype, using the PreemieNatalie simulator. As they did so, the Facilitators encouraged the participants to “Think Aloud” regarding their feedback and opinions regarding potential feasibility and acceptability of the device within the Kenyan setting, and to provide user-design feedback [51].

### Analysis

The audio and videotapes were translated, as required, and transcribed verbatim by a trained Kenyan research assistant who had not been present at the FGDs or KIIs and was fluent in both English and Kiswahili. In order to ensure accuracy of the transcription, immediately after the audio and videotapes were transcribed, one of the co-authors (SLB) reviewed the written transcripts and compared them to facilitator notes taken contemporaneously during FGDs and KIIs. Correction of minor transcription errors was performed as necessary, and notes were made about contextual factors related to each FGD or KII. De-identified transcripts were then provided to DM for additional review and any additional corrections or contextual notes. Subsequently, three co-authors (NAB; SL; SLB) engaged in iterative cycles of both independent and collective deductive and inductive coding followed by thematic content analysis. We utilized deductive methods to determine initial categories and themes (Cycle 1). Inductive methods were used to generate additional codes and sub-themes from written transcriptions (Cycle 2). We then employed thematic content analysis for finalization of themes and sub-themes, and to compare our study results with those from the general literature (Cycle 3). Investigator triangulation was extensively utilized during all phases of data analysis.

#### Literature review

The foundation for deductive coding was an extensive literature review and synthesis that was performed by NAB, SL, and SLB from May–August 2022. This literature review, which resulted in an annotated bibliography of 141 research products (e.g., peer-reviewed publications; presentations; dissertations or theses) was performed by accessing both published (PubMed) and gray (Google Scholar) literature. We downloaded, analyzed, and discussed papers regarding topics such as neonatal hypothermia and thermal care in LMICs; barriers, challenges, gaps, facilitators and enablers to KMC/STS; existing innovations, solutions, and biomedical devices to prevent and/or manage newborn hypothermia in both high- and low-resourced global regions. Results, including notes regarding each article that was analyzed during the literature review were tracked on a shared Excel spreadsheet. Information that was collated included: study citation; initials of the person populating the paper to the annotated bibliography; study design, methods, and outcomes reported; study aims or objectives; target population; country or global region where study was conducted; main findings and results; main themes, sub-themes; quality of the research product, as determined by checking the study according to the appropriate checklist or guideline for the study design (e.g., AMSTAR; CASP; COREQ, etc.).

#### Cycle 1: Independent deductive analysis to determine initial categories and themes

Upon completion of the literature review phase, analysis of the FGD and KII transcripts commenced. The researchers (NAB; SL; SLB) used an Excel spreadsheet template created specifically for this purpose to independently perform line-by-line assessments of each transcript and organize respondent comments into primary themes and categories. The purpose of this study was to elicit perspectives, from adult stakeholders of premature babies in Kenya, about the potential feasibility and acceptability of a novel biomedical device to prevent neonatal hypothermia and augment KMC/STS within the African setting. Suggestions were also elicited from these potential end-users regarding how to improve functions and features of the device. Thus, for deductive content analysis, it was decided *a priori* to organize each respondent’s answer within an interview or group discussion under one of three major themes–Feasibility, Acceptability, or User-design feedback. If the respondent’s answer did not match one of these themes or seemed to cross-cut more than one main theme, then it was assigned its own categorical code and flagged for discussion.

In order to ensure an adequate emerging rate of interrater reliability for deductive coding among the 3 researchers, after each coder had performed independent assessment of 3 transcripts, NAB, SL, and SLB met to compare their initial, on-going coding efforts via randomly selected samples. The researchers had perfect alignment on 31 of 36 randomly selected, independently coded samples (86% agreement). This included among 2 coders who had not been present at the FGDs/KIIs (NAB; SL), and 1 coder who had been a primary Facilitator of the FGDs/KIIs (SLB). These initial results provided greater confidence in the emerging alignment, among the 3 coders, regarding coding definitions and convergence of emerging categories and themes. Discrepancies in deductive coding were discussed and solved through adjudication. Coding definitions were further refined.

### Cycle 2: Content analysis through deductive and inductive coding

Upon confirmation of strong alignment in initial coding efforts, with methods adapted from Roberts et al., [2019; [52])] and Hsieh and Shannon [2005; [53]] each researcher utilized both deductive and inductive methods to independently generate additional categories and sub-themes and conduct content analysis (conventional and directed) for each transcript. Each researcher generated their own codebook, in Excel, with labels, descriptions, inclusions/exclusions, and examples, pulled from the raw text, of themes and sub-themes. After each researcher finished independently performing content analysis on the entire set of transcripts, the team met to compare their independently derived code books and discuss each theme and sub-theme that had emerged. Adjudication was applied to the results of the content analysis for each of the main themes (Feasibility, Acceptability, User Design Feedback), resulting in consolidation of some categories and sub-themes, and addition or expansion of others. Additional contextual information regarding the setting, participants, and transcripts was provided, as necessary, by SLB. A number of cross-cutting themes were collectively identified. By the end of this process, a single merged codebook, combining all 3 researchers’ collective inductive and deductive content analysis effort was generated.

### Cycle 3: Thematic analysis

The final cycle of qualitative analysis was conducted with methodology adapted from Crow, Inder, and Porter. [2015;[54]] Using the unified codebook and content analysis summation that resulted from Cycles 1 and 2, NAB and SL performed thematic analysis to ascertain how the themes and sub-themes identified in participant responses mapped to prior literature regarding barriers, challenges, gaps, bottlenecks, facilitators, and enablers to KMC/STS in LMICs. They also explored participant responses, and the emergent themes and sub-themes, as related to 3 research questions: (1) “Is the NeoWarm biomedical device feasible for facility-based KMC/STS in the Kenyan setting?” (2) “To what degree do Kenyan stakeholders agree to or approve/disapprove of the NeoWarm biomedical device?” and (3) “For the current NeoWarm biomedical device prototype, what has been done well and what can be done to improve the user’s experience?”

## Results

### Description of the study population

A total of 5 Focus Group Discussions (FGDs) and 17 Key Informant Interviews (KIIs) were held among 64 adult stakeholders (n = 38 women and 26 men) of premature babies aged 6 weeks or less (Table 1). This included among 30 parents or family stakeholders (n = 18 women and 12 men); 24 healthcare providers (n = 16 women and 8 men); 10 community opinion leaders (n = 4 women and 6 men).

**Table 1:** Number and type of adult stakeholders in Kenya who participated in NeoWarm feasibility, acceptability, and user-design feedback sessions.

	Parent or family member	Healthcare provider	Community opinion leader	Total
**Key Informant Interview**	0	17	0	**17**
**Focus Group Discussion (n)**	30 (3)	7 (1)	10 (1)	**47 (5)**
**Total**	**30**	**24**	**10**	**64**

### Theme 1: Feasibility of the NeoWarm biomedical device

Respondents from across stakeholder groups indicated that the NeoWarm device would be feasible within the Kenyan setting. Comments regarding feasibility clustered around sub-themes such as integration of the device into various KMC/STS contexts and affordability. Participants repeatedly identified that the NeoWarm device was feasible for both facility-based and community (home)-based KMC/STS.

#### Integration of NeoWarm into various KMC/STS contexts

“*Yes*, *actually it can be useful in the hospital especially in our scenario for example [facility without a KMC/STS ward]*. *Sometimes we have mothers who are upstairs and the baby lays down here*. *If the room is no warm enough or the baby is not covered well*, *at least they are likely to experience warmth [with the NeoWarm device]…*.*”*–Male nurse, tertiary referral center, KII

*“In fact it is good you teach the mothers to use it at home yeah*. *Maybe they forget that they are out of the hospital*, *everything is okay and they don’t continue with it*. *But since they have it*, *it is available and they know they know the importance*, *then they will use it*. *Once they are discharged maybe in the hospital maybe they go buy one*. *This is nice*. *It will encourage them to continue the kangaroo and that will strengthen the bond between the mother and the baby*, *the father and the baby*.*”*–Female nurse, tertiary referral center, KII

#### Affordability

The proposed price-point for the v1.01 prototype of the NeoWarm biomedical device was $20 - $25 USD, which at the time of the study, was equivalent to approximately 2000–2500 Kenyan shillings. Healthcare providers noted various potential direct and indirect cost benefits for integrating the NeoWarm device into facility-based KMC/STS care.

“*Affordable is the key*.”–Female Pediatrician, tertiary referral center, KII

“*Yeah*, *I think that is a good start for the price*, *and then given that it will be the hospital purchasing it*, *we can as well lobby for them [patients] to have*, *yeah*. *Yeah*, *it is practical*. *Because you realize the incubators cost at least 500*,*000 [$5000 USD]*, *and all of them have gone off [broken]*, *yeah*.”–Female KMC nurse, rural hospital with a KMC ward, KII

*“*…*Yeah*, *and even with the price you are talking about*, *most facilities can afford*. *We can afford*.*”*–Female nurse respondent, rural hospital without KMC ward, FGD

*“In a hospital*, *2000 [Kenyan shillings]*, *or is it too low 2000*? *Because I’m just trying to envision as much as KMC practice has tried to put down [reduce] the hospital length*, *you realize they [families] are being charged [hospital fees] every day*. *So if this mom stayed here for like thirty days eh*, *we are expecting at least she will part with 4000 shillings [$400 USD]*, *yes*.*”*–Female KMC nurse, rural hospital with a KMC ward, KII

There were mixed perspectives regarding affordability for individuals, especially those within rural settings.

“*For middle class person*, *it [price point of $20 - $25 USD] is ok*. *But for poor people in the interior*, *it won’t be possible*. *So they will have to use in the facility*. *It will really work in the facility*. *And those who can buy it are those ones who will stay with it at home*. *But the rural areas*, *it won’t help as such*, *because most of the people coming here [large tertiary referral hospital] are from the rural areas*. *And those are the people who are the ones that*, *they don’t go for clinics and whatever*, *and so they can encounter such things*. *Maybe for the facilities it will work*.*”*–Male nurse, tertiary referral hospital, KII

“*Yeah*, *looking at the rural set up*, *they can afford 2000 KES [$20 USD]*,*”*–Male respondent, Parent & family stakeholders, FGD

### Theme 2: Acceptability

Sub-themes related to acceptability emerged in regards to the device’s potential to reduce workload burden for healthcare providers and improve the delivery of newborn care interventions, particularly in regards to the frequency and accuracy of monitoring body temperature.

“*Yeah*, *actually if we are using the device*, *it would be a perfect*, *one in terms of relieving work load*, *and then another thing we will be very confident with the body temperature*. *You realize in our set up*, *at times it gets very cold*. *So yeah*, *even sometimes even we are forced to have a heater*, *yeah*. *And you know with the ordinary heater you can actually get the right temperatures of the baby*. *But now*, *ah with the NeoWarm*, *I think it will be a much better*. *We will be very confident with it that the baby’s temperature is right*.—Female KMC nurse, rural hospital with a KMC ward, KII

“…it will help us to reduce the workload that we have. Because if every mom would have this, you will only be to put the eyes across to see. Are they in uncomfortable positions, you read the colors [of the LED lights] if they are on, even if you are doing some other things, but even if you have five to six mamas, they will be comfortable and you can watch over them comfortably.”–Female nurse, rural hospital without a KMC ward, FGD

An unexpected sub-theme emerged in regards to respondents suggesting that NeoWarm is a potentially acceptable device to support neonatal transport, when babies are referred for higher-level care.

“In fact what you have said is very, very important. We have been getting babies who are brought here dying from hypothermia, and this one could be very important. So it could be one of these in an ambulance. And ambulance should be required. Maybe if they are carrying a neo or preterm, they can put them here because hypothermia is a major challenge.–Male nurse, tertiary referral center, KII

“And I was thinking during referrals, we have those small babies who are not stable. So they might need care in the NBU [newborn unit]…So I was thinking during referrals, it can be very handy in the facility because if the baby needs to be intubated? The baby is in sniffer position, and you can stretch the baby’s legs, and you can resuscitate as you refer. Contrary to the baby being on the mothers body, you can, where you can have issues in positioning the baby and doing resuscitation.”–Female nurse, rural hospital without a KMC ward, FGD

### Theme 3: User-design feedback

Four key sub themes emerged as a result of user design feedback sessions with key stakeholders—device comfort, safety, colors, and versatility. Many respondents reported that the device was comfortable to wear. Some requested that the device weigh less and suggested that the straps be reconfigured.

#### Comfort

*“Still this thing is very comfortable*. *The mother might relax and forget concerning the baby*.*”*–Female nurse, rural hospital with KMC ward, KII

*“Yeah*, *it’s good*. *Yeah*. *You can sit*, *you can lie*, *in a semi-seating position*, *but you will still feel comfortable”*–Female nurse, rural hospital without a KMC ward, FGD

*“This is very comfortable*. *The mothers are not going to want to give them up—and neither are the nurses*. *You have done a remarkable thing*. *Let’s get these going as soon as possible*, *to stop losing babies to hypothermia*.*”–*Male pediatrician, tertiary referral center, KII

*“I feel okay*. *Very comfortable*. *It is very okay*.*”*–Male respondent, Parent and family stakeholders, FGD

*“I wish it could be strapped differently*, *to maybe this side…And maybe*, *also about the straps*. *It should be coming with a*, *with a*, *maybe with an adjustable strap*.”–Female KMC nurse, rural hospital with KMC ward, KII

*“It is a bit heavy*, *I can feel it*. *Now the heavy part [battery pouch in front] is pulling me back*.–Male nurse, tertiary referral center, KII

*“I think it should be a bit lighter*.”–Female nurse, tertiary referral center, KII

#### Safety

Several stakeholders expressed how NeoWarm ensures confidence within the user that a baby will remain secure in the “flexed frog” position and not be at risk of slipping from the binder during KMC/STS.

*“The baby feels secure*.*”*–Female pediatrician, tertiary referral center, KII

*“It’s very comfortable*. *Its comfortable on the inside so you feel the baby is safe…it really helps*. *Do you know why*? *The mother is not worried of the baby falling down*. *The baby is well secured here and that one will give the mother time to concentrate on the baby*.*”*–Female nurse, tertiary referral center, KII

“*Yes*, *it is safe*. *I can even stand without*. *The baby can’t fall easily*.”–Male nurse, tertiary referral center, KII

“*You can cook*, *yeah*. *It will not restrict you from cooking and doing anything*, *you see*? *[mimes bending over to sweep and stir an imaginary pot of ugali]”*–Female respondent, Parent and family stakeholders, FGD

#### Colors

The v1.01 NeoWarm prototype had a gray carrier and purple swaddling pouch. In addition, a purple pocket on the outside of the carrier held the batteries and circuit board, protected by a 3D printed case. Respondents were asked their opinions about the current color configuration for the device, and whether they would prefer other color schemes.

“*…*.*I am for bright colors because me I like bright*, *and this is also for the baby*. *The baby will be comfortable in something brighter*, *yeah*. *Something flowery bright*.*”*–Female nurse, tertiary referral center, KII

*“These are male-friendly colors*.*”*–Male pediatrician, tertiary referral center, KII

“*But for this one*, *I don’t think you need to change the color*. *The color that you chose is okay*, *they are good colors*.”–Male nurse, tertiary referral center, KII

“*The colors are very ok*.”–Male respondent, Parent and family stakeholders, FGD

#### Versatility

Lasty, in regards to device versatility, some stakeholders suggested creating devices of various sizes and with different features, such creating a device to accommodate twins, various birthweights, and incorporating a wider range of vital signs monitoring. There were suggestions to create different forms of NeoWarm at various price points, with basic or more advanced features.

*“Yes*. *If it is possible maybe they manufacture two sizes*. *The one for the very*, *extremely low birth weight and another for moderate birth weight*. *If it is possible*. *That is*, *if these are babies without any other problems*.*”–*Female nurse, rural hospital with KMC ward, KII

*“So it would be also nice for the other aspects [automated vital signs monitoring and alarms] to be incorporated*. *So that when the baby’s airways would be compromised it would be announced*.*”–*Female KMC nurse, rural hospital with KMC ward, KII

*“For a hospital is affordable*. *I think it’s a good thing*, *but maybe we need different types*. *It’s like you have an SUV*, *which is fully loaded*, *and another one for those who could afford*.*”*–Female nurse, rural hospital with KMC ward, KII

#### Cross-cutting theme: Overcome traditional KMC/STS implementation challenges

A number of sub-themes were identified which combined elements of both feasibility and acceptability for the NeoWarm device, as related to the current paradigm of KMC/STS implementation in Kenya. Healthcare providers, parents, and community opinion leaders were aware of, and generally supportive of KMC/STS, but also reported numerous challenges related to consistent implementation of KMC/STS via traditional methods, which might be ameliorated via use of the NeoWarm biomedical device. These included difficulty in maintaining thermal care during intermittent KMC/STS, challenges related to use of traditional cloths (lesso/kanga cloth) as KMC binders, and gender role expectations.

#### Difficulties maintaining the thermal chain

“*Now we are having interrupted [intermittent] KMC; that would be the best thing*. *When you are having that baby by the side*, *when the mother feels like resting*, *that [stand-alone mode] will be the ideal thing for us to do*. *Because we will be sure that even the body temperature is optimized*. *Yeah*, *unlike when they are just put to the bedside eh*, *then the baby is cold*, *so we will not achieve the optimal [body] temperature*.”–Female nurse, rural hospital with KMC ward, KII

#### Challenges related to use of traditional cloths as KMC binders

Respondents reported numerous barriers to use of lessos/kanga cloths as KMC binders, including difficulty in tying them properly and safely positioning the baby, and that preterm births are often unexpected, leaving families unprepared with basic supplies such as 2 cloths.

“*Actually we are very much grateful for the KMC program*, *and we pray for the success of this new device*. *Running the lesso [for KMC/STS] takes much time but this [NeoWarm device] you can just put on… and then you just get out straight away*. *It actually is something that is good*. *We pray that it comes up very fast so that it is helpful for those people who are coming next”*–Male respondent, Community opinion leaders, FGD

*“It’s like kind of it [traditional kanga cloth] ties somebody around*, *it restricts somebody from most of the activities they are supposed to be doing*. *That’s one thing I know”*–Female nurse, rural hospital without KMC ward, FGD

*“I think*. *Okay*, *for my sake*, *I found it [NeoWarm device] okay*. *I find it okay*. *You realize with the traditional lessos*, *normally the mothers are advised to hold the baby’s head*. *At times eh*, *okay we normally wrap the earline*. *But when the baby is very active*, *it normally falls*. *So you keep on readjusting*. *But the beauty with this [NeoWarm] the baby is well placed*. *So it is just*, *my duty is just to check how the baby is breathing*.*”*–Female nurse, rural hospital with a KMC ward, KII

“That is the problem. Actually, the moms normally come without a wrapper. Now with preterm babies, these are babies that arrived too soon. These moms had not prepared for labor. So when they come in, they don’t have. Normally we even try to support them with the wrappers. So I think this, the NeoWarm is a perfect thing. It will save the mother from buying more wrappers, yeah. And the burden of washing. You even get the two that she is already having, they need to wash. So, the baby will be put off KMC until the wrapper is dry.–Female KMC nurse, rural hospital with KMC ward, KII

#### Gender role and sociocultural expectations

*“To fathers*, *I think this one will be more comfortable for them than tying the lessos*. *This one will be very much comfortable for them*. *It will look like a ragsack*. *They like carrying rags (laughter)*. *They can imagine it is that and they can carry it with all the comfort; its more preferable than the lessos*.*”*–Female nurse, rural hospital without a KMC ward, FGD.

*“This one will bring all sorts of people on board even men*. *I know men will accept with ease*. *You know if a colleague comes to my house and finds me in a lesso*, *and in the African context and the structure in Bungoma*, *you know a man is supposed to sit with visitors there*, *but not to have the baby*. *But with this I know now with the changing world around*, *it will get*, *it will be adopted*.”–Male nurse, rural hospital without KMC ward, FGD

“You know, according to our own African culture, it is the obligation of the mother [to provide KMC/STS]. But nowadays, men are participating in the process.”–Male nurse, tertiary referral center, KII

“They say the color is good and especially for men,”–Male respondent, Parent and family stakeholders, FGD

## Discussion

Biomedical devices have the potential to address barriers to KM/STSC in low-resource settings, yet only a handful of such innovations are described in the literature. One example is a specialized KMC wrap in Malawi that helped increase skin-to-skin contact in comparison to traditional binders.[55] Our group recently developed NeoWarm, a prototype for a self-heating and automated vital signs monitoring device that continuously monitors and regulates a newborn’s body temperature. The purpose of this study is to identify themes related to feasibility, acceptability, and user design of NeoWarm in the context of KMC implementation via qualitative analysis of focus group discussions and key informant interviews from various adult stakeholders of prematurely born infants in the Kenyan healthcare system. Below, we highlight some of the key findings from this qualitative study, in regards to adult stakeholders’ perspectives regarding the feasibility and acceptability of the NeoWarm device to overcome key barriers and challenges to KMC/STS, and facilitating KMC/STS, within the Kenyan setting.

### NeoWarm overcomes barriers to traditional KMC/STS

The qualitative data from key Kenyan stakeholders demonstrates that NeoWarm can be feasible in the context of implementation of facility-based KMC. Overall, respondents from the KIIs and FGDs appear to be enthusiastic and satisfied with the NeoWarm prototype.

#### For parents

Use of traditional cloths, such as *lesso* or kanga cloths in sub-Saharan Africa, and saris in Asian sites, can serve as a barrier to adoption, uptake, and compliance to KMC/STS. Often, women do not have multiple cloths available, and thus, when the traditional materials are soiled, or become wet, this interrupts KMC/STS until the one piece of fabric can be washed (often by hand), dried, and re-used. Several barriers to KMC/STS, related to use of traditional cloths as KMC binders, were identified by Kenyan participants. These included restricted movement (adult caregiver), lack of sufficient number of cloths to adequately support continuous KMC/STS, need to handwash and dry *lessos*, and the lack of available cloths in the context of unexpected preterm births. Adult stakeholders of premature infants in Kenya indicated that the NeoWarm biomedical device has the potential to address some of these challenges and barriers.

In agreement with what has also been reported by other investigators,[20] Kenyan respondents reported that safe positioning of the premature infant for KMC/STS can be a concern for both parent and healthcare provider stakeholders. Participants indicated that this concern was largely ameliorated by the design of the NeoWarm biomedical device.

Another major barrier to KMC uptake is traditional gender roles and expectations. Commonly, newborn care is a responsibility allocated to mothers,[22, 34] and a *lesso* is primarily associated with females only.[56] However, the interviews revealed that NeoWarm can potentially facilitate fathers becoming more involved in newborn care, particularly due to its lack of resemblance to a *lesso* and its “male-friendly” colors. Several nurses also provided recommendations for device colors, particularly emphasizing a desire for brighter, “baby friendly” colors.

One qualitative study in Ethiopia found that among patriarchal societies, a male’s perspective on newborn care may act as an enabler or barrier to the mother’s uptake of KMC.[57] Another qualitative study in Malawi found that KMC/STS can be burdensome and physically exhausting for mothers.[58] Kenyan participants in the current study revealed that NeoWarm has the potential to overcome gender role barriers in KMC/STS, thus potentially reducing some of the mother’s burden in newborn care by enhancing male involvement in the process of KMC/STS.

### For healthcare providers and health facilities

Poor infrastructure and inadequate logistics within healthcare facilities is a particularly challenging barrier to KMC/STS, since hospitals often do not have space, privacy, or a comfortable environment to support continuous KMC.[16, 19, 36, 59–62] In some settings, units such as the NICU have limited visitation policies that interfere with adult-baby dyads engaging in KMC/STS.[63] In contexts where lack of a formal KMC ward, or limited space, lack of privacy, or hospital policy limits mother and baby contact, Kenyan interviewees suggested NeoWarm can be a viable way to augment thermal care. In particular, respondents suggested that the device could help fill in gaps in intermittent KMC, via the stand-alone mode, when the mother needs to rest, and incubators are not available.

Healthcare financing continues to be a major bottleneck to dissemination of KMC/STS in low resource settings.[17] Financial buy-in from hospital administration is an important consideration for the successful implementation of KMC programs. Cost is another important component of a device’s feasibility. Interviewees described the proposed price point of the NeoWarm device as a feasible expense for health facilities and middle-class individuals, but potentially out of reach for rural communities. Some potential financial advantages to use of NeoWarm in health facilities, as outlined by healthcare worker respondents, included its low cost relative to the price of incubators.

**Other findings.** Respondents reported their perspective that NeoWarm is a feasible and acceptable device to support KMC/STS in both health facilities and the home (community) setting. Healthcare providers envisioned a system where mothers could be educated about and use the device in the facility setting then later purchase and use it at home. Per respondents, such a device, with its auto-heating and automated body temperature monitoring features, would give mothers the confidence that their babies would be adequately warmed while in the community setting. One healthcare worker described how utilization of NeoWarm, during the transition from hospital-based to home-based care, might reduce the frequency of outpatient follow-up visits, which is another benefit given that post-discharge follow-up for KMC is already challenging in low-resource settings.[21]

Another, unexpected respondent-identified use case was the use of NeoWarm in patient transport. Respondents who worked in a tertiary referral center noted that many infants who are referred from rural areas do not survive the transfer to higher-level care due to hypothermia.[27] Having a device such as NeoWarm in ambulances has the potential to reduce neonatal mortality and morbidity for referrals from distant rural areas where long-distance neonatal transport is very challenging due to poor road conditions and often associated with neonatal hypothermia and overall neonatal morbidity and mortality.[64]

## Limitations and strengths

This was a study conducted only within the setting of Kenya, among adult stakeholders in a limited geographical area. We employed purposive convenience sampling, rather than random sampling, Thus, the results are not necessarily generalizable to other settings or populations. We only conducted key informant interviews among healthcare providers, and not among parent or community stakeholders; thus, the opinions and perspectives of healthcare providers are likely overrepresented in this study. In part, this was purposeful, as the study was designed to explore the potential feasibility and acceptability of a novel biomedical device to support facility-based KMC/STS in LMICs. Although community opinion leaders and parents and family stakeholders of premature babies participated in focus group discussions, it is likely that, due to the nature of group dynamics versus one-on-one discussions, less in-depth information was captured from community and parental stakeholders. Including a local physician as a co-Facilitator was an overall strength of this study (see below), and key to the community participatory design strategy. However, it may have also influenced or biased participant’s responses. We believe this potential limitation was mediated by the fact that the local physician was not in a supervising role for any of the healthcare workers or community opinion leaders, nor was he the practicing physician for any of the babies whose parents participated in the study.

Strengths of the study included having a Kenyan co-Facilitator who is a pediatrician and fluent in Kiswahili. This allowed for participants to engage in either English or Kiswahili, whichever language they were more comfortable speaking. The KIIs and FGDs purposely integrated highly participatory and active user design feedback sessions, using a built prototype. Participants had ample opportunity to try on the device, using a neonatal premature baby simulator (manikin), and provide user-centered design feedback. For data analysis, investigator triangulation was extensively utilized, increasing potential trustworthiness of the results.

## Conclusions

Rich and detailed information extracted from interviews and focus group discussions with key stakeholder groups shows potential for NeoWarm to be acceptable and feasible in promoting adoption and uptake of KMC/STS in the Kenyan setting. Unexpected and novel findings derived from the participants include (1) the ability of the device to overcome several barriers to traditional KMC in LMICs and (2) the expansion of the use case of the device to include community or patient-transport settings. User design feedback obtained during the interviews will inform future improvements for the device.

## Supporting information

S1 Appendix Research team and reflexivity. (DOCX)

S2 Appendix Interview guide. (PDF)
