# Supplementary material for: Feasibility and acceptability of a novel biomedical device to prevent neonatal hypothermia and augment Kangaroo Mother Care in Kenya: Qualitative analysis of focus group discussions and key Informant Interviews
Source: PLOS Glob Public Health. 2024 Apr 16;4(4):e0001708. doi: 10.1371/journal.pgph.0001708 (PMC11020951; doi:10.1371/journal.pgph.0001708)
Supplement: S1 Appendix — (DOCX) [file pgph.0001708.s001.docx]

**S1 Research team and Reflexivity**

Drs. Sherri L. Bucher (SLB, female) and David Muyodi (DM, male) served as the co-PIs of the study. In this role, they both served as co-Facilitators for the KIIs and FGDs that were conducted in Kenya. SLB is a PhD-trained researcher with ample experience in global health and expertise in both quantitative[1-5] and qualitative research studies.[6-8] She currently serves as an Associate Professor and Chair, Department of Community and Global Health, Richard M. Fairbanks School of Public Health, as an Adjunct Associate Professor of Pediatrics within the Division of Neonatal-Perinatal Medicine at IU School of Medicine, as an Affiliate Faculty member of the IU Center for Global Health Equity, and as an IUPUI Translating Research into Practice Scholar. Dr Bucher has been a co-Investigator within the IU-Kenya Research Unit for the Global Network for Women’s and Children’s Health Research since 2008. From 2017 to 2023, she served as the US-based co-PI. Through her collaboration with the Global Network over the past 15 years, Dr. Bucher has worked closely with a variety of Kenyan colleagues, including DM on a wide variety of maternal-newborn-child health (MNCH) initiatives.[9-22] Previously, as an AMPATH co-investigator, she lived and worked in Kenya 2005 – 2006.[23] Currently, Dr. Bucher spends around 2 months a year on the ground in Kenya. This deep, lived experience within the Kenyan setting provides Dr. Bucher with rich knowledge regarding the challenges, barriers, gaps, facilitators, and opportunities to provision of high-quality newborn care, and implementation of evidence-based practices, within East Africa.

Dr. David Muyodi is a Kenyan physician who also holds a Masters’ of Public Health degree, and certification in Biomedical and Health Research Ethics. He has served as a Research Coordinator or co-Investigator on a number of important MNCH studies with integral community-engagement strategies, including the AFRINEST trial (community-based identification and treatment of neonatal sepsis),[24] FIRST LOOK (community-based ultrasound during pregnancy),[21, 25-29] and Helping Babies Breathe (neonatal resuscitation).[30] At the time in which this study was conducted, DM and SLB served as collaborators for the IU-Kenya Global Network site. In this manner, both DM and SLB have a deep understanding of the community and health facility contexts within the Kenyan setting. DM and SLB can be considered subject matter experts in regards to neonatal health and health systems within the Kenyan setting. This may have supported greater rigor for the construction of data collection tools (KII/FGD guide) and thematic analysis that was conducted as part of this study.

Dr. Muyodi is fluent English and Kiswahili, and, at the time this study was conducted, had established long-term partnerships with rural communities in western Kenya through his work on the AFRINEST and FIRST LOOK trials. The community relationships that SLB and DM have built, independently and collectively, with healthcare workers and communities, ensured the ability to recruit key adult stakeholder groups for inclusion in this study. The study participants were aware that SLB and DM were the co-Principal Investigators of the project. This information about their role, as well as contact information for both SLB and DM and a description of the purpose of the study, was provided on a study information sheet that was provided to the potential research subjects at recruitment, during the informed consenting process. In addition, during introductory remarks for each KII and FGD, Drs. Bucher and Muyodi formally introduced themselves to participants, described their role in the project, and reminded participants, in person, of the purpose of the project. The fact that SLB is a North American investigator, and DM is a Kenyan physician, may have influenced participants to respond in a more positive manner, during interviews and focus group discussions, due to a perceived imbalance in interpersonal power dynamics between the co-facilitators (DM and SLB) and study respondents. We tried to mitigate this risk by also inviting local Global Network Registry Administrators, who are embedded within the communities and have close collaborative relationships with parents and community opinion leaders, to participate in focus group discussions held among these stakeholder groups.

At the time this study was conducted, Nudar A. Bhuiya (NAB) and Scott Liu (SL) were 4^th^ year medical students at the Indiana University School of Medicine. From May 2020 – February 2023, they were participants of the competitively selected Scholarly Concentration in Public Health Certificate program, sponsored by the IUPUI Richard M. Fairbanks School of Public Health in Indianapolis. They were mentored by SLB to develop knowledge, skills, and competencies in qualitative analysis specific to this project. Under the mentorship of SLB, they performed literature review and data analysis. Under the joint tutelage of SLB and DM, they assumed primary responsibility for development of the current manuscript. Because the data analysis for this project was conducted, in part, by students as partial requirement to fulfil educational requirements, there might be threats to the rigor of the study. We tried to ameliorate this possibility via thorough, systematic training over a long period of time, highly engaged and structured hands-on mentoring (meetings at least once per month over the course of the project period) and following best practices for rigorous qualitative research.

SLB is the inventor of the NeoWarm biomedical device and holds intellectual property in regards to the device’s invention. She facilitated or co-facilitated some of the KIIs and FGDs reported in this paper. Thus, there is the potential threat of bias. The study team attempted to mitigate this possibility via several strategies. These included: 1. Semi-structured interview guides to standardize the interview and FGD facilitation process (S2 Interview Guide); 2. DM conducted a number of FGDs in Kiswahili, particularly in the rural communities – SLB was present at these sessions to answer questions from respondents, but is not a fluent Kiswahili speaker, so could not influence the interview process; 3. Investigator triangulation was extensively utilized during all phases of data analysis.

**S1 References**

1. Tita ATN, Carlo WA, McClure EM, Mwenechanya M, Chomba E, Hemingway-Foday JJ, et al. Azithromycin to Prevent Sepsis or Death in Women Planning a Vaginal Birth. New England Journal of Medicine. 2023. doi: 10.1056/NEJMoa2212111.

2. Bucher S, Marete I, Tenge C, Liechty EA, Esamai F, Patel A, et al. A prospective observational description of frequency and timing of antenatal care attendance and coverage of selected interventions from sites in Argentina, Guatemala, India, Kenya, Pakistan and Zambia. Reprod Health. 2015;12 Suppl 2(Suppl 2):S12. Epub 2015/06/13. doi: 10.1186/1742-4755-12-s2-s12. PubMed PMID: 26063483; PubMed Central PMCID: PMCPMC4464209.

3. Bucher S, Nowak K, Otieno K, Tenge C, Marete I, Rutto F, et al. Birth weight and gestational age distributions in a rural Kenyan population. BMC Pediatr. 2023;23(1):112. Epub 2023/03/09. doi: 10.1186/s12887-023-03925-2. PubMed PMID: 36890485; PubMed Central PMCID: PMCPMC9993805.

4. Bauserman M, Leuba SI, Hemingway-Foday J, Nolen TL, Moore J, McClure EM, et al. The efficacy of low-dose aspirin in pregnancy among women in malaria-endemic countries. BMC Pregnancy Childbirth. 2022;22(1):303. Epub 2022/04/12. doi: 10.1186/s12884-022-04652-9. PubMed PMID: 35399060; PubMed Central PMCID: PMCPMC8994890.

5. Hoffman MK, Goudar SS, Kodkany BS, Metgud M, Somannavar M, Okitawutshu J, et al. Low-dose aspirin for the prevention of preterm delivery in nulliparous women with a singleton pregnancy (ASPIRIN): a randomised, double-blind, placebo-controlled trial. Lancet. 2020;395(10220):285-93. Epub 2020/01/27. doi: 10.1016/s0140-6736(19)32973-3. PubMed PMID: 31982074; PubMed Central PMCID: PMCPMC7168353.

6. Bucher S, Konana O, Liechty E, Garces A, Gisore P, Marete I, et al. Self-reported practices among traditional birth attendants surveyed in western Kenya: a descriptive study. BMC Pregnancy Childbirth. 2016;16(1):219. Epub 2016/08/16. doi: 10.1186/s12884-016-1007-8. PubMed PMID: 27514379; PubMed Central PMCID: PMCPMC4981994.

7. Jewett CG, Sobiech KL, Donahue MC, Alexandrova M, Bucher S. Providing Emotional Support and Physical Comfort During a Time of Social Distancing: A Thematic Analysis of Doulas' Experiences During the Coronavirus Pandemic. Community Health Equity Research & Policy. 2022:0272684X221094172. doi: 10.1177/0272684X221094172.

8. Ray H, Sobiech KL, Alexandrova M, Songok JJ, Rukunga J, Bucher S. Critical Interpretive Synthesis of Qualitative Data on the Health Care Ecosystem for Vulnerable Newborns in Low- to Middle-Income Countries. J Obstet Gynecol Neonatal Nurs. 2021;50(5):549-60. Epub 2021/07/25. doi: 10.1016/j.jogn.2021.05.001. PubMed PMID: 34302768.

9. Bang A, Patel A, Bellad R, Gisore P, Goudar SS, Esamai F, et al. Helping Babies Breathe (HBB) training: What happens to knowledge and skills over time? BMC Pregnancy Childbirth. 2016;16(1):364. Epub 2016/11/24. doi: 10.1186/s12884-016-1141-3. PubMed PMID: 27875999; PubMed Central PMCID: PMCPMC5120476.

10. Bresnahan BW, Vodicka E, Babigumira JB, Malik AM, Yego F, Lokangaka A, et al. Cost estimation alongside a multi-regional, multi-country randomized trial of antenatal ultrasound in five low-and-middle-income countries. BMC Public Health. 2021;21(1):952. Epub 2021/05/22. doi: 10.1186/s12889-021-10750-8. PubMed PMID: 34016085; PubMed Central PMCID: PMCPMC8135981.

11. Garces A, McClure EM, Chomba E, Patel A, Pasha O, Tshefu A, et al. Home birth attendants in low income countries: who are they and what do they do? BMC Pregnancy Childbirth. 2012;12:34. Epub 2012/05/16. doi: 10.1186/1471-2393-12-34. PubMed PMID: 22583622; PubMed Central PMCID: PMCPMC3493311.

12. Garces A, McClure EM, Espinoza L, Saleem S, Figueroa L, Bucher S, Goldenberg RL. Traditional birth attendants and birth outcomes in low-middle income countries: A review. Semin Perinatol. 2019;43(5):247-51. Epub 2019/04/15. doi: 10.1053/j.semperi.2019.03.013. PubMed PMID: 30981470; PubMed Central PMCID: PMCPMC6591059.

13. Garces AL, McClure EM, Pérez W, Hambidge KM, Krebs NF, Figueroa L, et al. The Global Network Neonatal Cause of Death algorithm for low-resource settings. Acta Paediatr. 2017;106(6):904-11. Epub 2017/02/28. doi: 10.1111/apa.13805. PubMed PMID: 28240381; PubMed Central PMCID: PMCPMC5425300.

14. Harrison MS, Garces A, Figueroa L, Esamai F, Bucher S, Bose C, et al. Caesarean birth by maternal request: a poorly understood phenomenon in low- and middle-income countries. Int Health. 2021;13(1):63-9. Epub 2020/06/02. doi: 10.1093/inthealth/ihaa020. PubMed PMID: 32478383; PubMed Central PMCID: PMCPMC7807237.

15. Jessani S, Saleem S, Hoffman MK, Goudar SS, Derman RJ, Moore JL, et al. Association of haemoglobin levels in the first trimester and at 26-30 weeks with fetal and neonatal outcomes: a secondary analysis of the Global Network for Women's and Children's Health's ASPIRIN Trial. Bjog. 2021;128(9):1487-96. Epub 2021/02/26. doi: 10.1111/1471-0528.16676. PubMed PMID: 33629490; PubMed Central PMCID: PMCPMC8286300.

16. Leuba SI, Westreich D, Bose CL, Powers KA, Olshan A, Taylor SM, et al. Predictors of Plasmodium falciparum Infection in the First Trimester Among Nulliparous Women From Kenya, Zambia, and the Democratic Republic of the Congo. J Infect Dis. 2022;225(11):2002-10. Epub 2021/12/11. doi: 10.1093/infdis/jiab588. PubMed PMID: 34888658; PubMed Central PMCID: PMCPMC9159331.

17. Marete I, Tenge C, Chemweno C, Bucher S, Pasha O, Ramadurg UY, et al. Lost to follow-up among pregnant women in a multi-site community based maternal and newborn health registry: a prospective study. Reprod Health. 2015;12 Suppl 2(Suppl 2):S4. Epub 2015/06/13. doi: 10.1186/1742-4755-12-s2-s4. PubMed PMID: 26062899; PubMed Central PMCID: PMCPMC4464022.

18. McClure EM, Garces AL, Hibberd PL, Moore JL, Goudar SS, Saleem S, et al. The Global Network Maternal Newborn Health Registry: a multi-country, community-based registry of pregnancy outcomes. Reprod Health. 2020;17(Suppl 2):184. Epub 2020/12/02. doi: 10.1186/s12978-020-01020-8. PubMed PMID: 33256769; PubMed Central PMCID: PMCPMC7708188.

19. Patel AB, Bann CM, Kolhe CS, Lokangaka A, Tshefu A, Bauserman M, et al. The Global Network Socioeconomic Status Index as a predictor of stillbirths, perinatal mortality, and neonatal mortality in rural communities in low and lower middle income country sites of the Global Network for Women's and Children's Health Research. PLoS One. 2022;17(8):e0272712. Epub 2022/08/17. doi: 10.1371/journal.pone.0272712. PubMed PMID: 35972913; PubMed Central PMCID: PMCPMC9380930.

20. Shukla VV, Eggleston B, Ambalavanan N, McClure EM, Mwenechanya M, Chomba E, et al. Predictive Modeling for Perinatal Mortality in Resource-Limited Settings. JAMA Netw Open. 2020;3(11):e2026750. Epub 2020/11/19. doi: 10.1001/jamanetworkopen.2020.26750. PubMed PMID: 33206194; PubMed Central PMCID: PMCPMC7675108 the conduct of the study. Dr Hibberd reported grants from NIH during the conduct of the study. Dr Carlo reported personal fees from Mednax and serving on the company’s board of directors outside the submitted work. No other disclosures were reported.

21. Swanson DL, Franklin HL, Swanson JO, Goldenberg RL, McClure EM, Mirza W, et al. Including ultrasound scans in antenatal care in low-resource settings: Considering the complementarity of obstetric ultrasound screening and maternity waiting homes in strengthening referral systems in low-resource, rural settings. Semin Perinatol. 2019;43(5):273-81. Epub 2019/04/14. doi: 10.1053/j.semperi.2019.03.017. PubMed PMID: 30979599; PubMed Central PMCID: PMCPMC6597951.

22. Tikmani SS, Ali SA, Saleem S, Bann CM, Mwenechanya M, Carlo WA, et al. Trends of antenatal care during pregnancy in low- and middle-income countries: Findings from the global network maternal and newborn health registry. Semin Perinatol. 2019;43(5):297-307. Epub 2019/04/22. doi: 10.1053/j.semperi.2019.03.020. PubMed PMID: 31005357; PubMed Central PMCID: PMCPMC7027164.

23. Nyandiko WM, Otieno-Nyunya B, Musick B, Bucher-Yiannoutsos S, Akhaabi P, Lane K, et al. Outcomes of HIV-exposed children in western Kenya: efficacy of prevention of mother to child transmission in a resource-constrained setting. J Acquir Immune Defic Syndr. 2010;54(1):42-50. Epub 2010/03/13. doi: 10.1097/QAI.0b013e3181d8ad51. PubMed PMID: 20224420.

24. Tshefu A, Lokangaka A, Ngaima S, Engmann C, Esamai F, Gisore P, et al. Simplified antibiotic regimens compared with injectable procaine benzylpenicillin plus gentamicin for treatment of neonates and young infants with clinical signs of possible serious bacterial infection when referral is not possible: a randomised, open-label, equivalence trial. Lancet. 2015;385(9979):1767-76. Epub 2015/04/07. doi: 10.1016/s0140-6736(14)62284-4. PubMed PMID: 25842221.

25. Franklin HL, Mirza W, Swanson DL, Newman JE, Goldenberg RL, Muyodi D, et al. Factors influencing referrals for ultrasound-diagnosed complications during prenatal care in five low and middle income countries. Reprod Health. 2018;15(1):204. Epub 2018/12/14. doi: 10.1186/s12978-018-0647-8. PubMed PMID: 30541560; PubMed Central PMCID: PMCPMC6291965.

26. McClure EM, Nathan RO, Saleem S, Esamai F, Garces A, Chomba E, et al. First look: a cluster-randomized trial of ultrasound to improve pregnancy outcomes in low income country settings. BMC Pregnancy Childbirth. 2014;14:73. Epub 2014/02/19. doi: 10.1186/1471-2393-14-73. PubMed PMID: 24533878; PubMed Central PMCID: PMCPMC3996090.

27. Nathan R, Swanson JO, Marks W, Goldsmith N, Vance C, Sserwanga NB, et al. Screening obstetric ultrasound training for a 5-country cluster randomized controlled trial. Ultrasound Q. 2014;30(4):262-6. Epub 2014/11/22. doi: 10.1097/ruq.0000000000000096. PubMed PMID: 25415862; PubMed Central PMCID: PMCPMC4439948.

28. Nathan RO, Swanson JO, Swanson DL, McClure EM, Bolamba VL, Lokangaka A, et al. Evaluation of Focused Obstetric Ultrasound Examinations by Health Care Personnel in the Democratic Republic of Congo, Guatemala, Kenya, Pakistan, and Zambia. Curr Probl Diagn Radiol. 2017;46(3):210-5. Epub 2017/01/07. doi: 10.1067/j.cpradiol.2016.11.001. PubMed PMID: 28057388; PubMed Central PMCID: PMCPMC5413583.

29. Swanson JO, Plotner D, Franklin HL, Swanson DL, Lokomba Bolamba V, Lokangaka A, et al. Web-Based Quality Assurance Process Drives Improvements in Obstetric Ultrasound in 5 Low- and Middle-Income Countries. Glob Health Sci Pract. 2016;4(4):675-83. Epub 2016/12/30. doi: 10.9745/ghsp-d-16-00156. PubMed PMID: 28031304; PubMed Central PMCID: PMCPMC5199182.

30. Bellad RM, Bang A, Carlo WA, McClure EM, Meleth S, Goco N, et al. A pre-post study of a multi-country scale up of resuscitation training of facility birth attendants: does Helping Babies Breathe training save lives? BMC Pregnancy Childbirth. 2016;16(1):222. Epub 2016/08/17. doi: 10.1186/s12884-016-0997-6. PubMed PMID: 27527831; PubMed Central PMCID: PMCPMC5477802.
