# Supplementary material for: Feasibility and acceptability of a novel biomedical device to prevent neonatal hypothermia and augment Kangaroo Mother Care in Kenya: Qualitative analysis of focus group discussions and key Informant Interviews
Source: PLOS Glob Public Health. 2024 Apr 16;4(4):e0001708. doi: 10.1371/journal.pgph.0001708 (PMC11020951; doi:10.1371/journal.pgph.0001708)
Supplement: S2 Appendix — (PDF) [file pgph.0001708.s002.pdf]

S2 Appendix: NeoWarm Feasibility and Acceptability FGD and KII guide

Instructions for NeoWarm FGD/KII Facilitators:

1. Greet potential participants.

2. Introduce yourself, briefly explain the general purpose of the NeoWarm device ("*To prevent hypothermia among small and preterm babies, and augment skin-to-skin care*"), and explain the purpose of the FGD or KII ("*We are interested in your thoughts and feedback regarding whether you think this device will be feasible and acceptable in the African setting. We are also interested in your feedback about how to make the design of this device better. Finally, we are very interested in your thoughts about whether or you believe this device is safe—another way to ask is, we are interested in your feedback regarding whether you believe this device could potentially harm babies or mothers. If so, we will ask your opinion about what you would do to reduce or eliminate that potential for harm, and make this device more safe.*")

3. Request that potential participants review the Study Information Sheet. Encourage them to ask any questions or clarifications along the way. Allow potential participants to take their time reading the sheet. Do not rush them. If a stakeholder is of low or no literacy, offer to read the SIS sheet to them; ensure that they understand each section. If the stakeholder is more comfortable speaking in Kiswahili, then provide translation of the SIS. Upon completion of the SIS review, if they verbally re-consent to participate in the FGD/KII, allow them to do so in Kiswahili.

4. If the NeoWarm stakeholder re-consents to participate in the study, ensure that they are also aware that the FGD and/or KII may be audio or videotaped. Ask if they agree to this.

    a. If the stakeholder agrees to participate in the study, and agrees to be audio and/or videotaped, have him/her sign and date the SIS. Facilitator also signs and dates the SIS.

    b. If the stakeholder agrees to participate in the study but does not wish to be audio and/or videotaped, please make a note of this directly on their SIS. **Do not audio or videotape this person as they provide feedback.** Take notes by hand.

    c. If the stakeholder, upon review of the SIS, does not wish to participate in the FGD or KII, thank him/her for his/her time, and politely excuse them from the FGD/KII.

NeoWarm FGD/KII Guide

Before commencing:

--If participants have given permission to be video and/or audiotaped, turn on the recording device.

--State the date, place, and what type of FGD or KII is being held (for example: *"We are at MTRH with a group of 6 newborn health care providers, who have agreed to provide their opinions and feedback about the NeoWarm biomedical device. The date is March 21, 2016. I am the Facilitator, Dr. Sherri Bucher*.)

--Then, confirm that all have consented to be recorded: *"I just want to confirm that you have all read, understood, and signed the Study Information Sheet, and that you have agreed to have your feedback recorded by video and/or audiotape—is this correct?"*

--Ensure that participants agree verbally before proceeding.

--If using an audio recording device, demonstrate to each of the participants how to use the audio recorder before you begin the FGD or KII.

FGD/KII prompts regarding the current landscape for newborn hypothermia

1. Do you believe that it is dangerous for newborn babies (those babies from birth to 1 month of age) to become cold? Why or why not?
2. What causes newborn babies to become cold?
3. In your experience, is keeping newborn babies warm, those who are 1 month or less, difficult? Why or why not?
4. Currently, how do you know if a newborn baby is warm or cold? Babies can't use words to tell us that they are cold—how do you, as an adult, know when a newborn is cold?
5. What are the methods you currently use to keep newborn babies warm?
6. When you detect that a baby is cold, and then try to warm it, using the methods you've described, how do you know that the methods you've used have been successful (i.e., how can you tell that the baby is now warm, and not cold)?
7. Would you describe the current methods that you use to keep babies warm as effective? Why or why not?
8. Are there any populations of babies which seem to have a harder time staying warm?
    a. If so, what are the methods you use to keep these babies warm—same or different from other babies?
9. With the method(s) of keeping babies warm that that you currently use, are there any times when there are "thermal gaps," and the baby is losing warmth? (e.g., during diaper changes; bathing; when mother takes personal hygiene breaks; during patient care procedures)

General questions regarding knowledge, attitudes, and perceptions toward skin-to-skin/Kangaroo Mother Care

10. Have you heard of “skin-to-skin” care or kangaroo mother care? If so, what are your thoughts about skin-to-skin/KMC? Is it good? Bad? Neutral?
11. In your experience, if you have some experience with skin-to-skin/KMC, did you find it to be effective? Why or why not?
12. What do you think is the biggest advantage of skin-to-skin/KMC? Biggest disadvantage?
13. In your experience, what are some barriers, or challenges, to skin-to-skin care/KMC?
14. Do you consider skin-to-skin care/KMC to be “hard or “difficult?” For whom? Why?

****INTRODUCE THE NEOWARM PROTOTYPE. FACILITATOR DEMONSTRATES THE STAND ALONE AND KMC FUNCTIONS USING THE PREEMIE NATALIE NEWBORN SIMULATOR****

General questions regarding the device

15. What are your immediate thoughts/impressions about this device? Please be very frank, and share with us both your “positive” and “negative” feedback.
16. If you were to simply see the NeoWarm device lying on a table, and did not know its function, would you classify it as a “feminine,” “masculine,” or “gender-neutral” object?
17. What is your understanding of the purpose of this device?
18. When you see this device, what are your thoughts about using it in a health facility setting?
    a. Do you think that it would be beneficial? Why or why not?
19. What are your thoughts about use of this device in the community setting?

****ASK: Does anyone here know how to do traditional skin-to-skin/KMC? Would you be willing, with this Preemie Natalie simulator and *lesso* (provided), to demonstrate for us how to bind a baby in the traditional KMC/skin-to-skin manner?” Have 2 of the participants demonstrate traditional binding for skin-to-skin/KMC with the *lesso*. Then, have these same participants use NeoWarm in the skin-to-skin/KMC mode. Provide the NeoWarm User’s Guide so that they can use the pictures and text to guide them on how to don/doff the device.******

ASK: Would all of you like a chance to compare the traditional vs. NeoWarm KMC binding options?

For those participants who have given permission, VIDEO TAPE THIS PORTION: Allow participants to go in pairs, and assist each other with trying the traditional and NeoWarm methods. This includes male participants!

When everyone has had a chance to try both methods, say: We have now all had a chance to try both the traditional skin-to-skin binding and the NeoWarm skin-to skin binding methods. I am going to ask your feedback and opinions about these 2 methods:

20. What is most different about NeoWarm compared to a KMC binder? Is this a good or bad change?
21. Which method do you believe is *easier to use* (put on and take off)? Why?
22. Which method did you, as the *adult wearer*, find more comfortable? Why?
    a. Did the NeoWarm device feel "light," "heavy," or in-between?
23. Do you think the *baby* would feel more comfortable in the traditional KMC binder or NeoWarm? Why?
24. Did the "baby" feel equally secure (you did not fear the mannequin would slip out of the binder) with both binding methods?
25. An important component of KMC is facilitation of breastfeeding. With which KMC binding method, traditional or NeoWarm, do you think it will be easier for mothers to breastfeed? Why?
26. Do you think that it would be easier to keep NeoWarm or the traditional binder clean and dry? Why?
27. Some small or premature babies require "continuous" skin-to-skin care/KMC, which is defined as the baby being skin-to-skin with an adult caregiver for 20 or more hours per day. What are your thoughts regarding whether you, or other stakeholders, might prefer a traditional KMC binder methods or NeoWarm for "continuous" KMC?
28. Have you ever seen a man perform skin-to-skin/KMC?
29. What if you went to a health facility, and saw a father doing KMC with a traditional binder—what would you think? What if you saw him wearing NeoWarm?
30. What if you saw a man in the community doing KMC with a traditional binder? What is you saw him wearing NeoWarm?
31. If you went to visit someone in a health facility, and saw a mother using NeoWarm with their baby, what would you think? If you saw a mother in the community wearing NeoWarm, what would you think?
32. Are there other persons for whom NeoWarm might be appropriate? Grandparents? Aunties/Uncles? Older siblings?
33. Please describe to me what you understand to be the function of these colored lights.
34. Do you believe that the colored lights are useful? Why or why not?
    a. If you consider the lights to be useful, to whom are they most useful—mothers? Health care providers? Family stakeholders? All of the above?

   b. Where do you believe the lights should be positioned on the device to be most beneficial?

35. We have developed an "auditory alarm" feature to go with the colored lights. There are different tones that go with the 'red' and the 'blue' lights. Do you think that this is a useful feature?
   a. If you believe it is useful to have auditory alarms, for whom are they most useful—mothers? Health care providers? Family stakeholders? All of the above?
   b. How loud do you believe the alarms should be to be the most useful?
36. Please tell me your thoughts about the "pouch" (purple) portion of the device. Do you have any concerns about it—for example, do you think it will fit babies properly? Will it keep babies warm during the "stand alone" function? What would you do to improve it?
37. If NeoWarm were available today, would you use it for your own baby? Would you recommend it to your neighbors?
38. Does anyone have any final comments, thoughts, concerns, or feedback about the NeoWarm device?

SAY: "*Thank you so much for taking the time to participate in this discussion today, and to share your thoughts, opinions, and feedback about how to keep babies warm. The information you provided to us today is incredibly useful, and we are very grateful for your assistance.*"

*****END of FGD/KII for family stakeholder and community opinion leader participants*****

Additional questions for healthcare providers

39. Do you think mothers will be more or less likely to use NeoWarm (vs traditional KMC binder)? Why?
40. What are your thoughts about the feasibility and acceptability of NeoWarm among male stakeholders, for use by their female partners?
41. What are your thoughts regarding the potential for NeoWarm to increase male stakeholders' OWN involvement in skin-to-skin care/KMC—in your opinion, would males themselves be more or less willing to engage in KMC using NeoWarm?
42. What are the environmental factors, or other challenges encountered in your health facilities, that might impact the effectiveness of NeoWarm, especially in regards to keeping babies warm?
43. As compared to traditional KMC methods, do you think that NeoWarm will help or hinder your daily work flow? Why?
   a. Are there specific ways that you anticipate NeoWarm could make your work easier or harder?

44. What functional components would you like to see added/removed in future iterations of NeoWarm device? (e.g., monitoring of vital signs)
45. In general, do you think NeoWarm is potentially more or less safe for small and premature babies, as compared to traditional methods of KMC (or are they equal)? Why?
46. Do you see any ways in which adult clients might misuse NeoWarm, in a way that can cause harm either to themselves or the baby?
47. Please be frank in sharing any potential concerns you have about NeoWarm. Do you see potential harm from this device, to mothers, babies, and/or family stakeholders?
    a. In what ways can we work to mitigate these potential harms?
48. In general, to you feel that NeoWarm would be a barrier to implementation of KMC programs in health facilities? Why or why not?
49. Do you believe that NeoWarm can serve as a facilitator for KMC programs in health facilities? How?
50. Do you believe that NeoWarm might serve as a barrier to incubator care in health facilities? Why or why not?
51. If NeoWarm was being manufactured today, and approved for use with babies, would you recommend that NeoWarm be incorporated into skin-to-skin/KMC efforts at your health facility? Why or why not?

SAY: Thank you so much for taking the time to participate in this discussion today, and to share your thoughts, opinions, and feedback about how to keep babies warm. The information you provided to us today is incredibly useful, and we are very grateful for your assistance.

*****END of FGD/KII for health care provider participants*****
